# Identifying Host-Characteristics and Management Risk Factors in a California Equine Herpesvirus Myeloencephalopathy (EHM) Outbreak

**DOI:** 10.3390/vetsci13020121

**Published:** 2026-01-27

**Authors:** Shadira Gordon, Nicola Pusterla, Carrie J. Finno, Amy Young, Beatriz Martínez-López

**Affiliations:** 1 Department of Medicine and Epidemiology, School of Veterinary Medicine, University of California, Davis, CA 95616, USA; dsgordon@ucdavis.edu (S.G.); npusterla@ucdavis.edu (N.P.); 2 Department of Population Health and Reproduction, School of Veterinary Medicine, University of California, Davis, CA 95616, USA; cjfinno@ucdavis.edu; 3 Center for Equine Health, School of Veterinary Medicine, University of California, Davis, CA 95616, USA; ayoung@ucdavis.edu

**Keywords:** equine herpesvirus-1 (EHV-1), equine herpesvirus myeloencephalopathy (EHM), epidemiology, biosecurity

## Abstract

Equine alphaherpesvirus-1 (EHV-1) is a highly contagious virus that can cause the neurological form, equine herpesvirus myeloencephalopathy (EHM). Understanding transmission-related risk factors is crucial for improving prevention strategies and guiding effective control measures. In this study, we collected data from 63 horses that had previously participated in the February 2022 winter horse show season at the Desert International Horse Park (DIHP) (26 cases and 37 controls) to identify host and management factors associated with EHV-1 infection and/or EHM development during the February 2022 outbreak at the DIHP in Thermal, California. Risk factors were evaluated using multivariable logistic regression and a random forest model with conditional permutation importance. Greater age was associated with higher odds of becoming a case (OR = 1.33; 95%CI: 1.04–1.69, *p*-value: 0.01). Compared with hunters, jumpers had greater odds of developing EHV-1 and/or EHM (OR = 7.37; 95%CI: 1.57–34.61, *p*-value: 0.01). Sharing a barn was also strongly associated with EHV-1 and/or EHM case status (OR = 7.37; 95%CI: 1.79–30.29, *p*-value: <0.01). The machine-learning-based rankings were concordant with the regression estimates. Age, main activity, and sharing a barn were the most influential risk factors associated with elevated odds of developing EHV-1 and/or EHM. These results highlight specific demographic and management-related risk factors that could inform targeted prevention strategies.

## Simple Summary

Despite extensive efforts to mitigate the spread of equine alphaherpesvirus-1 and equine herpesvirus myeloencephalopathy, including new vaccination strategies and treatment alternatives, the frequency of outbreaks reported worldwide has been increasing. The objectives of this study were to identify the main risk factors associated with the 2021–2022 outbreak of equine herpesvirus myeloencephalopathy in Thermal, California, and provide recommendations to mitigate the risk of future outbreaks. Sixty-three questionnaires regarding host demographics, biosecurity, and diet management were obtained. We identified that increasing age, main activity (jumpers vs. hunters), and sharing a barn with horses from different home barns were associated with higher odds of becoming a case. These findings provide useful insights for refining biosecurity protocols against equine alphaherpesvirus-1 and equine herpesvirus myeloencephalopathy, particularly for horses that routinely participate in equestrian events.

## 1. Introduction

Equine alphaherpesvirus-1 (EHV-1) was first isolated in the U.S. in 1933 from an abortion case [1,2], with the associated clinical manifestations extending to respiratory disease, neonatal death, conjunctivitis, and neurological deficits [3,4]. The neurological manifestation of EHV-1 is referred to as equine herpesvirus myeloencephalopathy (EHM) [5].

As a multifactorial disease, different components play important roles in its pathogenesis. Age, sex, and breed have been identified as specific risk factors for EHM [6]. Recent outbreak investigations have revealed higher odds of developing EHM in recently vaccinated horses, while other analyses suggest no effect or fewer EHM cases among vaccinated horses [7,8,9]. Although vaccination may still reduce virus shedding and is sometimes recommended in the face of outbreaks, its specific effects on EHM risk remain uncertain [10]. Consequently, prevention relies on recognizing who is at highest risk and how management practices can amplify exposure.

Even with substantial interventions aimed at limiting EHV-1 spread, it has been estimated that around 15% to 27% of the global equine population is latently infected with EHV-1 [10], while some studies have reported an estimated prevalence of 80 to 90% by the time horses reach two years of age [11,12,13].

In recent years, outbreaks of EHM have increasingly been reported worldwide, with the EHV-1 outbreaks in California from 2021 to 2023 causing the cancellation of several equine competitions. According to data from the International Federation for Equestrian Sports (FEI), California hosts the second-highest number of international equestrian events per year in the U.S., and boasts a substantial equine population, being surpassed only by Texas [14]. It receives a significant number of horses from diverse origins, contributing to an increased vulnerability to potential outbreaks of EHV-1 and EHM due to the extensive movement and interaction among horses [15,16].

In this study, we aimed to describe the epidemiological characteristics of the 2021–2022 EHV-1 outbreak at the Desert International Horse Park (DIHP) in Thermal, California; identify risk factors associated with EHV-1 and/or EHM status; and generate customized recommendations to mitigate the risk of future EHV-1 disease outbreaks.

## 2. Materials and Methods

### 2.1. Study Design

An unmatched case–control study design was implemented, and questionnaires were collected using a non-random sampling method (i.e., convenience sampling) between November 10th and 17th, 2022. Horses were selected based on their previous participation in the 2021–2022 winter horse show season at the DIHP, held from February 2nd to February 22nd, 2022. The questionnaire included previously reported determinants of EHV-1 [8,9,17,18,19,20,21,22], subclassified into host demographics, biosecurity, and diet management items (Appendix A).

From the initial set of 43 items, we retained 15 candidate questions (Table 1). Questions were excluded from further analysis according to the following criteria: (i) identifier fields, (ii) items used in the case definition, (iii) near-zero variance (i.e., imbalance ≲ 5% or ≳95% in one level), and/or (iv) >10% missingness. Variables excluded due to near-zero variance included vaccination status (all horses in the study were vaccinated), health history (survival, concurrent disease, previous EHV-1 infection), biosecurity practices during the event (isolation, quarantine, shared water source), and post-diagnosis management (barn disinfection, isolation, and testing after diagnosis).

To reduce sparsity and improve interpretability, three diet-related items (steamed/soaked hay, oils in diet, high-grain diet) were combined into a single “diet management” variable (Appendix B). The responses from the questionnaires were cross-referenced by the DIHP management and veterinarians, when applicable. The dataset that supports the findings of this study is available from the corresponding author upon reasonable request.

Horses with fever, detection of EHV-1 via qPCR (QuantStudio 5 Real-Time PCR System; Applied Biosystems, Thermo Fisher Scientific, Waltham, MA, USA) in blood and/or nasal secretions, and/or clinical disease (i.e., lethargy, anorexia, limb edema, respiratory signs) were classified as EHV-1-positive. Horses with neurological deficits (i.e., incoordination, recumbence, inability to get up, head tilt, urinary incontinence) and detection of EHV-1 via qPCR in blood and/or nasal secretions were classified as EHM positive. To preserve statistical power and maintain a balanced case-control ratio, we classified horses as cases if they were EHV-1 and/or EHM-positive, consistent with the approach used in a prior outbreak investigation [8]. Controls were defined as horses without clinical signs and with negative EHV-1 qPCR results in blood and/or nasal secretions.

Due to limitations in participant availability and the use of convenience sampling, only 63 individuals were enrolled (26 cases and 37 controls). This sample size was sufficient to detect an odds ratio of 10, assuming a case–control ratio of 1:1.5, with 80% power and a significance level of 0.05 [23].

### 2.2. Statistical Approach

First, we employed a directed acyclic graph (DAG) to guide the selection of confounders. DAGs provide a transparent, visual framework for encoding prior knowledge about the relationships between variables, allowing researchers to identify a minimally sufficient adjustment set that blocks confounding pathways while avoiding inappropriate adjustment for mediators or colliders. Importantly, while DAGs are rooted in causal inference theory, we used this approach strictly for principled variable selection; as such, we do not intend to imply causality from the findings of this observational case–control study. Second, given the limited sample size and the occurrence of perfect separation in our data, we applied a bias correction method to the multivariable logistic regression model. This penalized likelihood approach yields finite, less-biased parameter estimates when traditional maximum likelihood estimation fails to converge or produces implausibly extreme odds ratios. Third, we complemented the regression analysis with conditional permutation importance (CPI) derived from a random forest model. This nonparametric method allows for assessment of variable importance without assuming linearity or a specific functional form, providing independent validation of the variables identified through the DAG-guided regression approach.

### 2.3. Directed Acyclic Graph (DAG) Gold Standard Change in Estimate Procedure

**Figure 1:**
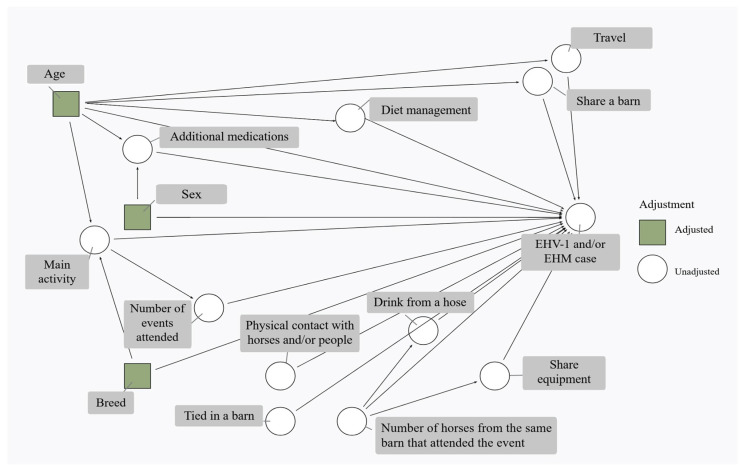
Directed acyclic graph of EHV-1 risk factors. The directed acyclic graph (DAG) represents the underlying causal structure assumptions between the exposures of interest and EHV-1/EHM development. Green boxes represent a minimally sufficient set of variables for confounding control.

A minimally sufficient set of confounders and conditional independence relations between the observed variables and the potential outcome were illustrated using a DAG (Figure 1) with the R Statistical Software (v4.5.2; R Core Team 2025) and the webtool “DAGitty” [24,25].

Among the variables included, the smallest set that blocked all biasing paths between the main exposure of interest (main activity) and the outcome (EHV-1 and/or EHM) using the backdoor criterion included sex, breed, and age, represented as green squares in Figure 1.

The DAG gold standard change-in-estimate (CIE) approach for variable selection was implemented using the SAS software version 9.4 (SAS Institute Inc.) [26], according to prior literature [27]. The initial full model included age, sex, and breed as potential confounding variables; the main exposure of interest: main activity; and the remaining 10 covariates. Each covariate was retained in the multivariable logistic regression model (Table 2) if the change in the odds ratio (Δ*OR*) after removing that variable was higher than 10% according to the following equation:

(1) $$∆\mathrm{OR}\;=\;\frac{\left(\left|ORi-OR\;\mathrm{DAG}\right|\right)}{\left(OR\;\mathrm{DAG}\right)}$$

where

*ORi* = odds ratio for the *i*th model

*OR* DAG = odds ratio from the initial full DAG model.

**Table 2:** DAG gold standard change-in-estimate procedure. Factors and OR estimates for variables with more than 10% change in the OR are presented in bold.

Model	Factor	OR Estimate	% Change OR
A	- Additional medications	17.69	4.07
B	- Number of events attended	19.13	3.94
C	- **Tied in a barn**	**15.05**	**18.21**
D	- Drink from a hose	20.16	9.5
E	- **Physical contact**	**21.23**	**15.31**
F	- **Share equipment**	**15.60**	**15.26**
G	- **Share barn**	**10.91**	**40.71**
H	- **Travel**	**22.02**	**19.65**
I	- Number of horses from the same barn	20.01	8.69
J	- Diet management	16.65	9.5

Multicollinearity was assessed according to the variance inflation factor (VIF) calculated using the R software [28] for each of the predictors included in the multivariable logistic regression model (Table 3).

### 2.4. Multivariable Logistic Regression Model with Bias Correction Approach

Due to perfect separation and limited sample size concerns, a bias correction approach [29] was considered over traditional methods (e.g., exact logistic regression) to evaluate the associations between the variables of interest and EHV-1 development in the multivariable logistic regression model. The bias correction method enables convergence to finite estimates and, therefore, provides a bias of smaller asymptotic order than that of the maximum likelihood estimator [30]. This results in a better estimate of the relationship between the risk factors and the outcome, considering the sample size, complete separation, and sparse data, while providing an interpretation that is equal to those obtained through traditional methodologies [31]. The “brlgm” package in R was used to incorporate the bias correction approach [28].

### 2.5. Conditional Permutation Importance 

**Figure 2:**
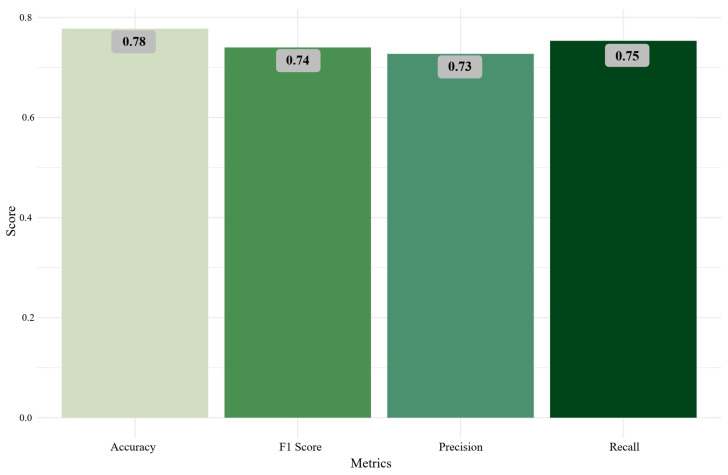
Random forest model performance indicators.

A nonparametric conditional permutation importance (CPI) approach using a random forest model was implemented to compare the results obtained from the DAG gold- standard change-in-estimate (CIE) approach. The CPI approach ranks the importance of the variables of interest regarding EHV-1 and/or EHM development [32]. The importance of the covariates was measured to classify a horse as a case or as a control conditioned on the remaining covariates. The model performance indicators included accuracy, F1 score, precision, and recall (Figure 2).

A risk factor was considered important depending on how much the model relied on the variable for the prediction of a case or a control, in addition to the remaining predictors; in particular, a risk factor was considered unimportant if the model did not change after changing the values, and the variable was ignored for making the prediction of a case or a control.

The application of the conditional permutation importance method provides a powerful means to discern those variables that exert the greatest influence on the outcome and to identify those that may contribute through interactions with other predictors [32].

## 3. Results

The questionnaires were completed by the trainers and/or owners, through 56 face-to-face interviews, 6 via telephone, and 1 online (the trainer was not in the U.S.). None of the individuals included in the study reported a positive EHV-1 test before the time of the outbreak.

A total of 26 EHV-1 and/or EHM cases were identified during the outbreak. All the horses included in this study had been vaccinated against EHV-1, with most receiving their most recent vaccination within one to four months prior to the outbreak. Cases tended to be slightly older than controls, with a mean age of approximately 12 years compared with 10 years among controls. The sex distribution was similar in both groups, with geldings comprising the majority in both. Ponies and horses competing as jumpers were more frequently represented among cases, whereas warmbloods and hunters were more common among controls. The most notable difference between groups was sharing a barn at the event, with cases more likely to have shared a barn with other horses than controls. Other potential exposure factors, such as physical contact between horses, shared equipment use, and drinking from a communal hose, were similarly distributed between cases and controls.

### 3.1. DAG Change-in-Estimate Approach

To guide variable selection for the multivariable model, a directed acyclic graph (DAG)-based CIE approach was applied. Based on this method, the following variables were included in the multivariable logistic regression model: age, sex, breed, main activity, being tied in a barn, physical contact, share equipment, share a barn, and travel (Table 4).

### 3.2. Multivariable Logistic Regression Model with Bias Correction Approach

For every additional year of age, there were 33% higher odds of becoming a case (OR = 1.33; 95%CI: 1.04–1.69, *p*-value: 0.01). When the main activity was characterized as show jumpers, there were 7.37 times greater odds of developing EHV-1 and/or EHM compared with those whose main activity was hunters (OR = 7.37; 95%CI: 1.57–34.61, *p*-value: 0.01). Sharing a barn, defined as horses from different home facilities being housed within the same barn structure at the show venue, was also a significant risk factor to developing clinical EHV-1 or EHM infection, where horses sharing a barn had 7.37 times greater odds of becoming cases compared with those that did not share a barn (OR = 7.37; 95%CI: 1.79–30.29, *p*-value: <0.01).

### 3.3. Random Forest Model Using Conditional Permutation Importance

**Figure 3:**
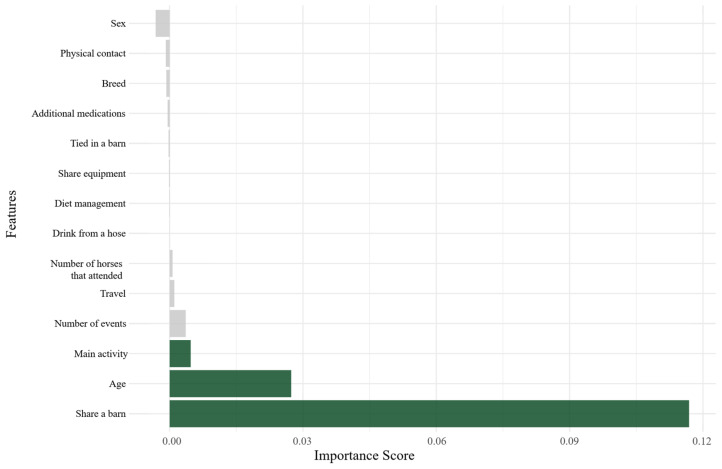
Conditional permutation importance approach measuring the degree to which the accuracy of the model increases or decreases when risk factors are randomly removed.

The variables that contributed the most to the predictions made by the random forest model are shown in Figure 3. Sharing a barn was the most influential predictor, improving the accuracy by 10% when retained in the model relative to when it was permuted, followed by age (2.5%) and main activity (2%).

## 4. Discussion

For this study, we designed a questionnaire encompassing host characteristics, biosecurity measures, and nutritional management strategies previously described in the literature [6,8,10] to investigate potential risk factors associated with EHV-1 transmission during the 2021–2022 winter horse show season from February 2nd to February 22nd, 2022, at the DIHP in Thermal, California.

All horses included in this study had been vaccinated against EHV-1, with most receiving their most recent dose within one to four months prior to the outbreak. As the vaccination status had no variability in our study population, we were unable to evaluate it as a risk factor. The occurrence of this outbreak among fully vaccinated horses is consistent with the known limitations of current EHV-1 vaccines. While vaccination may reduce the severity of clinical signs and decrease viral shedding, it does not reliably prevent infection or the development of EHM [10,20].

Given these limitations, identifying additional host and management factors associated with the risk of infection remains important.

A retrospective analysis of 13 outbreaks across 3 European countries identified increasing age and female sex as risk factors for higher EHM incidence, as determined by a logistic mixed model [9]. These findings align with those of a four-year retrospective study conducted in the Netherlands, where older and female horses were more likely to develop severe neurologic disease [33]. Similarly, during a recent outbreak in Monufia, Egypt [34], and an EHM outbreak at the University of Findlay’s English riding complex [35], horses older than five years were found to have significantly higher odds of EHV-1 infection and neurologic disease or death, when compared with those aged under one year. In the study in Egypt, males were twice as likely to become infected [34]. Conversely, an epidemiological study of the multistate EHM outbreak originating in Ogden, Utah, reported that younger horses were more susceptible to disease, while female sex remained a consistent risk factor [8]. These contrasting results reflect differences in study populations, outbreak settings, circulating viral strains, case definitions, and/or the demographic composition of horses at each event, highlighting the importance of localized epidemiological studies to better understand the dynamics of EHV-1 transmission and inform targeted control and prevention strategies. The present investigation represents one of the first detailed outbreak characterizations in California and contributes valuable insights regarding risk factors specific to showground horse events.

Age, main activity (jumpers versus hunters), and sharing a barn with horses from different home barns were statistically significantly associated with the odds of EHV-1 and/or EHM case status (*p*-value < 0.05). Age has been consistently identified as a significant factor influencing susceptibility to EHV-1 infection in horses [17,18]. In our study, cases were older (mean 11.8 years) than controls (mean 9.8 years), which generally aligns with findings from past outbreaks [9,34,35] but contrasts with the Utah multistate outbreak, where younger horses were reported to be more susceptible [8]. Older horses have increased odds of developing equine herpesvirus myeloencephalopathy (EHM), with studies estimating that each additional year of age may elevate this risk by approximately 6% [9]. This age-associated vulnerability is further supported by findings that mares over 20 years of age are more likely to develop EHM, a trend consistent with the concept of immune senescence observed in aging horses [6,36,37]. Unlike previous studies in which female sex was identified as a consistent risk factor [8,9,33], we did not observe a significant difference in sex distribution between cases and controls, with geldings representing the majority of both groups. This discrepancy may reflect differences in the sex of horses typically participating in hunter/jumper competitions. Previous studies reporting female sex as a risk factor were frequently conducted in breeding populations or mixed-use facilities where intact mares are more prevalent [8,9,33]. The predominance of geldings in our study population may have limited our ability to detect sex-based differences in susceptibility.

While age and sex are key individual risk factors, environmental and management practices also play a critical role in disease transmission. EHV-1 primarily spreads through direct horse-to-horse contact, respiratory droplets, and indirectly via contaminated equipment, feed, or water buckets [38,39]. The findings of the present study reinforce the idea that shared environments can facilitate the spread of EHV-1, likely due to insufficient implementation of biosecurity measures. The questionnaire in this study addressed specific risk factors, including the sharing of water buckets and equipment. However, future research should examine additional potential sources of environmental contamination, including barn proximity and ventilation systems, to better understand transmission dynamics. This premise aligns with findings from the CES Valencia Spring Tour 2021 outbreak, where inadequate ventilation was identified as a potential factor contributing to increased exposure to aerosolized virus shed by infected horses [21].

Stress may also increase susceptibility and viral reactivation in latently infected horses. Pre-competition management practices aimed at minimizing stress responses, such as individualized training schedules and optimized rest periods, are potential strategies for reducing viral reactivation.

However, while all horses experienced similar stressors associated with travel and environmental changes, the nature of athletic demand differs substantially between disciplines. Horses in jumper classes are judged on their speed and accuracy, which may partially explain the higher stress levels in these horses compared with those in hunter classes, which are judged on their style, manners, and movement [40,41]. This distinction parallels findings in human athletes, where competition itself, rather than shared environmental stressors, appeared to trigger symptomatic infection in previously asymptomatic individuals [42]. Whether the high-intensity demands of show-jumping classes contribute to transient immune modulation in horses warrants further investigation; especially considering that stress is a well-established trigger for EHV-1 reactivation in latently infected horses, potentially increasing the odds of transmission [1,13,41,43,44,45].

Given the early onset of latent infection in horses [12,13] and the disproportionate burden of EHM among older individuals [6,37], biosecurity protocols tailored to reduce exposure and stress in this high-risk cohort are strictly necessary.

Decision-makers should consider implementing practices such as limiting direct contact between high-risk horses, reducing proximity among horses in shared barns, and enhancing biosecurity protocols. Owners, veterinarians, trainers, and event organizers can apply the results identified in this study to reduce the spread of EHV-1 and EHM development, particularly in equine populations involved in competitive activities.

Several limitations should be considered when interpreting these findings. As with most observational studies, residual confounding from unmeasured variables cannot be excluded despite our use of the DAG-CIE approach. Additionally, the retrospective collection of questionnaire data may have introduced recall bias, as respondents aware of their horse’s disease status may have systematically recalled or reported exposures differently than those with unaffected horses. As the questionnaires were completed by trainers and riders rather than veterinary professionals, this may have led to variable interpretation of clinical signs. Although the responses were cross-referenced with the DIHP management and veterinary staff when available, some degree of misclassification cannot be excluded. Similarly, management practices at equestrian facilities are often correlated (e.g., facilities implementing one biosecurity measure may be more likely to implement other measures), which may limit our ability to isolate the independent contributions of individual factors. While the sample size was sufficient to detect large effect sizes (odds ratio ≥ 10) with 80% power [23], the study may have been underpowered to detect moderate associations, and therefore, the non-significant findings should be interpreted with caution.

Although horses were classified as EHV-1 positive based on the presence of fever, qPCR detection in blood and//or nasal secretions, and/or compatible signs, not all horses were tested via qPCR. Consequently, co-infections or alternative etiologies, such as equine influenza virus, cannot be entirely excluded in animals presenting with respiratory signs alone. Recognizing the challenges posed by a limited sample size, an integrative methodological framework combining multivariable logistic regression and random forest modeling was implemented to assess the potential risk factors related to EHV-1 and EHM. The DAG-CIE procedure has been identified as a convenient approach for binary outcomes when using a case–control study design, as it can yield a reduced standard error and provide better precision even in DAGs that include non-confounders [25]. Furthermore, this approach outperforms stepwise methods by maintaining stability even when omitted confounders are present [30].

Similarly, a random forest model with a conditional permutation importance approach was included, which is particularly effective for evaluating correlated predictors in datasets with small sample sizes [31]. The most important variables ranked in the conditional permutation approach included main activity, age, and sharing a barn. The results obtained from this supervised learning method were consistent with the results obtained from the multivariable logistic regression model, where age, main activity, and sharing a barn were statistically significantly associated with becoming an EHV-1 and/or EHM case.

The use of convenience sampling may have led to selection bias. Future prospective studies would benefit from stratified sampling approaches (e.g., by event class, barn location, or origin facility) for improved representativeness and generalizability. However, such designs may be challenging to implement during active outbreak investigations where participant availability is limited, and timely data collection must be prioritized. Alongside these methodological improvements, future research should also move beyond these associations and explore underlying biological mechanisms, such as immunological factors that influence host susceptibility, as well as the specific virulent factors involved in disease progression.

Even though the small sample size limited further model adjustments, resulted in wide confidence intervals, and precluded the evaluation of interaction effects between variables, this study highlights the multifactorial nature of EHV-1 and EHM and addresses a critical gap in the understanding of EHV-1 and EHM risk factors within high-performance equine populations, particularly in competitive environments where stress, close contact, and biosecurity challenges converge. As EHV-1 continues to pose a significant threat to equine health and the stability of the industry, this study provides actionable insights for equine welfare, offering a foundation for broader studies to integrate these findings into a wider framework to benefit diverse equine populations.

## Figures and Tables

**Table 1:** Characteristics of EHV-1 and/or EHM cases and controls.

	Controls (*N* = 37)	Cases (*N*= 26)	Total (*N* = 63)
Age			
Mean (SD)	9.81 (3.23)	11.80 (2.95)	10.63 (3.24)
Median (IQR)	10 (5.00)	12 (4.75)	11 (5.00)
Sex			
Gelding	27 (73.0%)	20 (76.9%)	47 (74.6%)
Mare	10 (27.0%)	6 (23.1%)	16 (25.4%)
Breed			
Warmblood	25 (67.6%)	13 (50.0%)	38 (60.3%)
Ponies	12 (32.4%)	13 (50.0%)	25 (39.7%)
Main activity			
Hunter	27 (73.0%)	10 (38.5%)	37 (58.7%)
Jumper	10 (27.0%)	16 (61.5%)	26 (41.3%)
Additional medications: steroids, progestins or other medications 6 months before attending the event and/or at the time of the outbreak			
No	26 (70.3%)	18 (69.2%)	44 (69.8%)
Yes	11 (29.7%)	8 (30.8%)	19 (30.2%)
Number of events per year before the event			
Mean (SD)	6.35 (8.38)	7.08 (7.04)	
Median [Min, Max]	2.00 [0, 25.00]	5.00 [0, 25.00]	
Number of horses attended from the same barn			
Mean (SD)	15.41 (6.60)	17.2 (10.8)	
Median [Min, Max]	15.0 [4.00, 27.0]	13.5 [6.00, 42.0]	
Tied in a barn			
No	34 (91.9%)	22 (84.6%)	56 (88.9%)
Yes	3 (8.1%)	4 (15.4%)	7 (11.1%)
Drink from a hose			
No	26 (70.3%)	20 (76.9%)	46 (73.0%)
Yes	11 (29.7%)	6 (23.1%)	17 (27.0%)
Physical contact			
No	13 (35.1%)	7 (26.9%)	20 (31.7%)
Yes	24 (64.9%)	19 (73.1%)	43 (68.3%)
Shared equipment			
No	17 (45.9%)	11 (42.3%)	28 (44.4%)
Yes	20 (54.1%)	15 (57.7%)	35 (55.6%)
Shared barn			
No	33 (89.2%)	11 (42.3%)	44 (69.8%)
Yes	4 (10.8%)	15 (57.7%)	19 (30.2%)
Travel			
No or within the U.S.	24 (64.9%)	20 (76.9%)	44 (69.8%)
International	13 (35.1%)	6 (23.1%)	19 (30.2%)
Diet management: steamed/soaked hay and/or supplements consumption			
No	7 (18.9%)	7 (26.9%)	14 (22.2%)
Yes	30 (81.1%)	19 (73.1%)	49 (77.8%)

**Table 3:** Variance inflation factor (VIF) for each predictor included in the final model.

Variable	VIF Value
Age	1.39
Sex	1.38
Breed	1.17
Main activity	1.46
Tied in a barn	1.18
Physical contact	1.30
Share equipment	1.38
Share a barn	1.09
Travel	1.33

**Table 4:** Multivariable logistic regression model with bias correction. Odds ratios, CIs and *p*-values for the associations of age, sex, breed, main activity, being tied in a barn, physical contact, share equipment, sharing a barn, and travel with EHV-1 and/or EHM development.

	OR	CI Lower Limit	CI Upper Limit	*p*-Value
Age (in years)	1.33	1.04	1.69	0.01
Sex				
Gelding (reference)				
Mare	1.53	0.28	8.28	0.61
Breed				
Warmblood (reference)				
Ponies	1.87	0.47	7.36	0.36
Main activity				
Hunter (reference)				
Jumper	7.37	1.57	34.61	0.01
Tied in a barn				
No (reference)				
Yes	2.80	0.23	33.60	0.41
Physical contact				
No (reference)				
Yes	0.96	0.21	4.37	0.96
Share equipment				
No (reference)				
Yes	1.84	0.41	8.27	0.42
Share a barn				
No (reference)				
Yes	7.37	1.79	30.29	<0.01
Travel				
No or within the U.S. (reference)				
International	1.59	0.33	7.59	0.55

## Data Availability

The raw data supporting the conclusions of this article will be made available by the authors on request.

## Abbreviations

The following abbreviations are used in this manuscript:

EHV-1	Equine alphaherpesvirus-1
EHM	Equine herpesvirus myeloencephalopathy
DIHP	Desert International Horse Park
FEI	International Federation for Equestrian Sports
qPCR	Quantitative polymerase chain reaction
DAG	Directed Acyclic Graph
CIE	Change in estimate

## Appendix A. Questionnaire “Assessment of the Risk Factors for EHV-1 Infection in Horses at the Desert International Horse Park”

Section I—Demographic characteristics

1. Horse name
2. FEI ID number
3. USEF ID number
4. Sex:
  ▢ Gelding
  ▢ Mare

5. Age (years)
6. Breed:
  ▢ Warmblood
  ▢ Pony
  ▢ Other, please specify.

7. What is the main activity of this horse?
  ▢ Hunter
  ▢ Jumper
  ▢ Other, please specify.

8. Was your horse confirmed to be infected with EHV-1? (Y/N/Unknown)
9. Was your horse vaccinated for EHV-1 before attending the DIHP? (Y/N/Unknown)
10. Please provide the date(s), product(s), and manufacturer(s) used to vaccinate this horse. *If necessary, please contact your veterinarian to obtain this information*
11. What is the current status of the horse?
  ▢ Alive
  ▢ Euthanized/Died due to EHV-1 infection at DIHP
  ▢ Euthanized/Died from other causes than EHV-1. If so, please specify the possible/confirmed cause of death and date (mm/dd/yyyy).

Section II—Clinical information

12. Date of confirmed EHV-1 diagnosis during DIHP event (mm/dd/yyyy)
13. Did the horse exhibit any of the following clinical signs? Please choose all that apply:
  ▢ Fever (Adult horses with a body temperature > 101.0 °F|38.3 °C)
  ▢ Lethargy/Lack of energy
  ▢ Anorexia/Lack of appetite
  ▢ Limb edema/Leg swelling
  ▢ Respiratory signs (nasal discharge, cough)
  ▢ Neurologic deficit (incoordination, weakness, recumbence, inability to get up, head tilt, urinary incontinence)

14. What was the body temperature and date in which fever was first recorded? Please specify if it was recorded in °F or °C.
15. Once diagnosed with EHV-1, was the horse isolated from other equids on the premise? (Y/N/Unknown)
16. Once diagnosed with EHV-1, the horses in the same barn were quarantined? (Y/N/Unknown)
17. Did any additional horses stabled in the same barn develop clinical EHV-1? (Y/N/Unknown)
18. Has this horse returned to its previous performance level? (Y/N/Unknown)
19. Was the horse receiving corticosteroids, anti-inflammatories, altrenogest (Regumate) or any other drugs before and/or during its attendance at the DIHP? (Y/N/Unknown)
20. Please list the product name, frequency, and date(s) in which it was provided. *If necessary, please contact your veterinarian to obtain this information*
21. Did the horse have any concurrent disease (excluding EHV-1) between 11th February 2022–13th March 2022? (Y/N/Unknown)
22. Please specify the name of the disease, date of diagnosis, and treatment implemented.

Section III—Biosecurity measures

23. Other than Thermal, in how many events did the horse compete in 2021?
24. Please specify the date and event name.
25. In how many classes did the horse compete during Thermal?
26. Which of the following apply to the horse during its stay in Thermal?
  ▢ Tied in a barn outside of a stall
  ▢ Share water source
  ▢ Drink from a bucket of unknown source
  ▢ Grazed
  ▢ Utilized a wash rack
  ▢ Had physical contact with other personnel (e.g., veterinarian, farrier, groom, braider) or horses
  ▢ Share hay nets, scrubs, stall webbings, or any other equipment/cloth
  ▢ Share barn with horses from different farms

27. Please provide the barn number in which your horse was located. *If necessary, refer to the facility map below to identify the correct number*
28. Was the barn disinfected during the horse stay in Thermal? (Y/N/Unknown)
29. Was the vehicle in which the horses arrived disinfected before attending Thermal? (Y/N/Unknown)
30. Was the horse previously (before DIHP) infected with EHV-1? (Y/N/Unknown)
31. In the previous 12 months before the event, did the horses from your facility travel? If so, please specify:
  ▢ Facilities within the U.S.
  ▢ Facilities outside the U.S.
  ▢ Events, shows, races inside the U.S.
  ▢ International events
  ▢ Veterinary hospital
  ▢ Does not apply

32. On average, how many horses are stabled at your home facility?
33. How many of the horses stabled at your home facility attended Thermal?
34. After attending an event, which biosecurity protocols are routinely implemented at your facility? Please choose all that apply:
  ▢ Cleaning and disinfection of equipment and trailer that went to the show
  ▢ Isolating horses that participated in an event
  ▢ Monitoring rectal temperature daily for horses returning from a show event
  ▢ Not sharing equipment (e.g., feeding, grooming, riding equipment) between resident horses and horses returning from the event
  ▢ EHV-1 testing for the horses that returned from an event
  ▢ Others, please specify.

Section IV—Management

35. How was the horse kept at your home facility during each season of the year previous to the outbreak in Thermal?

	**Spring**	**Summer**	**Fall**	**Winter**
Outdoors	▢	▢	▢	▢
Partly outdoors/partly stabled	▢	▢	▢	▢
Entirely stabled	▢	▢	▢	▢

36. What other animal species were in contact with your horse while attending Thermal? Please choose all that apply:
  ▢ Dogs
  ▢ Cats
  ▢ Birds
  ▢ Others, please specify.

37. How was your horse grouped at the event in Thermal? Please choose all that apply to your horse:
  ▢ Age
  ▢ Use
  ▢ Location of the facility
  ▢ Feeding needs
  ▢ Trainer
  ▢ No specific groups
  ▢ Others, please specify.

38. Did your home facility experience any infectious disease outbreak prior attending Thermal? (e.g., strangles, influenza, etc.) (Y/N/Unknown)
39. Please specify the name of the disease, date, and if applicable, the corrective actions implemented to prevent recurrence.
40. Was the horse under a high-energetic grains and/or oils diet 6 months before or during its attendance at the DIHP? (Y/N/Unknown)
41. Please specify the time period in which the diet was implemented.
42. Did the horse consume steamed/soaked hay 6 months before or during its attendance at the DIHP? (Y/N/Unknown)
43. Please specify the average time period of consumption.

## Appendix B. Questionnaire Variables and Data Reduction Rules

**Variable Name**	**Identifier Fields**	**>10% Missingness**	**Used in Case Definition**	**Near Zero Variance**	**Grouped with Other Variable(s) and New Variable Name**
1. Subject	Yes				
2. Club	Yes				
3. Case Control	Yes				
4. Horse name	Yes				
5. Equestrian Canada number	Yes				
6. FEI number	Yes				
7. USEF number	Yes				
8. Age					
9. Sex					
10. Breed					
11. Main activity					
12. EHV-1					Yes (outcome variable: EHM and/or EHV1)
13. Vaccinated				Yes	
14. Vaccination date		Yes			
15. Vaccine manufacturer		Yes			
16. Vaccine name		Yes			
17. Survival				Yes	
18. EHM					Yes (outcome variable: EHM and/or EHV1)
19. Lethargy			Yes		
20. Neurologic signs			Yes		
21. Respiratory signs			Yes		
22. Fever			Yes		
23. Fever date		Yes			
24. Isolated				Yes	
25. Quarantined				Yes	
26. Additional cases				Yes	
27. Previous level			Yes		
28. Time recovery days			Yes		
29. Additional medications					
30. Medication name		Yes			
31. Medication dose		Yes			
32. Medication frequency		Yes			
33. Concurrent disease				Yes	
34. Number of events attended					
35. Classes	Yes				
36. Tied in a barn					
37. Share water				Yes	
38. Drink from a hose					
39. Physical contact with other personnel and/or horses					
40. Share equipment					
41. Share barn					
42. Barn number	Yes				
43. Barn disinfection				Yes	
44. Vehicle disinfection		Yes			
45. Previous infection				Yes	
46. Travel					
47. Travel date		Yes			
48. Travel location		Yes			
49. Number of horses from the same barn that attended the event					
50. Barn disinfection after diagnosis				Yes	
51. Isolation after diagnosis				Yes	
52. Monitoring temperature after diagnosis					
53. Testing after diagnosis				Yes	
54. Horse housing				Yes	
55. Contact with other species				Yes	
56. Grouped category	Yes				
57. Supplements				Yes	
58. Type of supplements				Yes	
59. High grain diet					Yes (variable: Diet management)
60. Oils diet					Yes (variable: Diet management)
61. Steamed hay					Yes (variable: Diet management)
